# Association of Melanoma-Risk Variants with Primary Melanoma Tumor Prognostic Characteristics and Melanoma-Specific Survival in the GEM Study

**DOI:** 10.3390/curroncol28060401

**Published:** 2021-11-16

**Authors:** Danielle R. Davari, Irene Orlow, Peter A. Kanetsky, Li Luo, Klaus J. Busam, Ajay Sharma, Anne Kricker, Anne E. Cust, Hoda Anton-Culver, Stephen B. Gruber, Richard P. Gallagher, Roberto Zanetti, Stefano Rosso, Lidia Sacchetto, Terence Dwyer, David C. Gibbs, David W. Ollila, Colin B. Begg, Marianne Berwick, Nancy E. Thomas

**Affiliations:** 1 Department of Dermatology, University of North Carolina, Chapel Hill, NC 27599, USA; danielle_davari@med.unc.edu; 2 Department of Epidemiology and Biostatistics, Memorial Sloan Kettering Cancer Center, New York, NY 10065, USA; orlowi@mskcc.org (I.O.); Ajay.sharma2@perkinelmer.com (A.S.); beggc@mskcc.org (C.B.B.); 3 Department of Cancer Epidemiology, Moffitt Cancer Center and Research Institute, Tampa, FL 33612, USA; peter.kanetsky@moffitt.org; 4 Department of Internal Medicine, University of New Mexico Cancer Center, University of New Mexico, Albuquerque, NM 87102, USA; lluo@salud.unm.edu (L.L.); mberwick@salud.unm.edu (M.B.); 5 Department of Pathology, Memorial Sloan Kettering Cancer Center, New York, NY 10065, USA; busamk@mskcc.org; 6 Sydney School of Public Health, The University of Sydney, Sydney, NSW 2006, Australia; anne.kricker@sydney.edu.au; 7 Daffodil Centre, The University of Sydney, a Joint Venture with Cancer Council NSW, Sydney, NSW 2006, Australia; anne.cust@sydney.edu.au; 8 Melanoma Institute Australia, The University of Sydney, Sydney, NSW 2065, Australia; 9 Department of Medicine, University of California, Irvine, CA 92697, USA; hantoncu@uci.edu; 10 City of Hope National Medical Center, Duarte, CA 91010, USA; sgruber@coh.org; 11 BC Cancer and Department of Dermatology and Skin Science, University of British Columbia, Vancouver, BC V5Z 1L3, Canada; rgallagher@bccrc.ca; 12 Piedmont Cancer Registry, Centre for Epidemiology and Prevention in Oncology in Piedmont, 10156 Turin, Italy; roberto.zanetti@cpo.it (R.Z.); stefano.rosso@cpo.it (S.R.); lidia.sacchetto@cpo.it (L.S.); 13 Murdoch Children’s Research Institute, Melbourne, VIC 3052, Australia; terence.dwyer@wrh.ox.ac.uk; 14 The Nuffield Department of Women’s & Reproductive Health, University of Oxford, Oxford OX3 9DU, UK; 15 Department of Pediatrics, University of Melbourne, Melbourne, VIC 3010, Australia; 16 Oxford Martin School, University of Oxford, Oxford OX1 3BD, UK; 17 School of Medicine, Emory University, Atlanta, GA 30322, USA; david.corley.gibbs@emory.edu; 18 Department of Surgery, University of North Carolina, Chapel Hill, NC 27599, USA; david_ollila@med.unc.edu; 19 Lineberger Comprehensive Cancer Center, University of North Carolina, Chapel Hill, NC 27599, USA

**Keywords:** melanoma, single nucleotide polymorphism, Breslow thickness, ulceration, mitoses, tumor-infiltrating lymphocytes, survival

## Abstract

Genome-wide association studies (GWAS) and candidate pathway studies have identified low-penetrant genetic variants associated with cutaneous melanoma. We investigated the association of melanoma-risk variants with primary melanoma tumor prognostic characteristics and melanoma-specific survival. The Genes, Environment, and Melanoma Study enrolled 3285 European origin participants with incident invasive primary melanoma. For each of 47 melanoma-risk single nucleotide polymorphisms (SNPs), we used linear and logistic regression modeling to estimate, respectively, the per allele mean changes in log of Breslow thickness and odds ratios for presence of ulceration, mitoses, and tumor-infiltrating lymphocytes (TILs). We also used Cox proportional hazards regression modeling to estimate the per allele hazard ratios for melanoma-specific survival. Passing the false discovery threshold (*p* = 0.0026) were associations of *IRF4* rs12203592 and *CCND1* rs1485993 with log of Breslow thickness, and association of *TERT* rs2242652 with presence of mitoses. *IRF4* rs12203592 also had nominal associations (*p* < 0.05) with presence of mitoses and melanoma-specific survival, as well as a borderline association (*p* = 0.07) with ulceration. *CCND1* rs1485993 also had a borderline association with presence of mitoses (*p* = 0.06). *MX2* rs45430 had nominal associations with log of Breslow thickness, presence of mitoses, and melanoma-specific survival. Our study indicates that further research investigating the associations of these genetic variants with underlying biologic pathways related to tumor progression is warranted.

## 1. Introduction

Genome-wide association studies (GWAS) and candidate pathway studies have identified low-penetrant genetic variants associated with cutaneous melanoma [1,2]. Previously we investigated the association of 47 single nucleotide polymorphisms (SNPs) in putative melanoma-risk loci identified through GWAS or candidate studies with multiple primary melanoma occurrence and found that several of these susceptibility loci are generalizable to the risk of subsequent melanomas [3]. Many of these variants are in gene regions associated with pigmentation, such as *SLC45A2*, *TYRP1*, *TYR*, and *ASIP* [4,5,6,7,8,9,10,11]; nevi, such as *NID1*, *MTAP*, and *PLA2G6* [4,6,12,13,14,15,16,17,18]; or both, such as *IRF4* and *HERC2/OCA2* [4,7,13,14,19,20,21,22,23]. Others are in gene regions, including *ATM* and *MX2*, not associated with melanoma-risk phenotypes [5]. Variants related to pigmentation and/or nevus count variation likely modify melanoma risk via these mechanisms, while others may modify risk via alternative mechanisms, such as cell proliferation [5,24].

To explore whether genetic variants associated with melanoma risk could influence tumor aggressivity, we examined the associations of melanoma-risk SNPs with primary melanoma tumor prognostic characteristics. Prognostic characteristics in melanoma include Breslow thickness, ulceration, mitoses, and tumor-infiltrating lymphocytes (TILs). Breslow thickness and ulceration are the primary melanoma tumor characteristics included in the eighth edition of the American Joint Committee on Cancer staging system [25]. The presence of mitoses and a lower TIL grade are associated with worse melanoma-specific survival [26,27,28,29,30]. We assessed the association of melanoma-risk SNPs with log of Breslow thickness, presence of ulceration, presence of mitoses, and presence of TILs in the large, international, population-based Genes, Environment, and Melanoma (GEM) Study. To investigate whether genetic variants associated with melanoma risk could influence outcomes, we also examined the associations of these SNPs with melanoma-specific survival.

## 2. Materials and Methods

### 2.1. Study Population

The GEM Study enrolled 3579 participants with incident first- or higher-order primary cutaneous melanoma diagnosed between 1998 and 2003 in Australia, Canada, Italy, and the United States; recruitment and data collection details have been published previously [31]. Each recruitment site’s institutional review board approved the study. Participants provided written informed consent. Of the 3579 patients, we limited analyses to the 3285 participants of self-reported European origin with invasive first- or higher-order primary melanoma. Twelve participants of non-European origin were excluded. An additional 282 patients with incident in situ melanoma were also excluded, as Breslow thickness, ulceration, mitoses, and TIL presence are not relevant for in situ melanomas. Thus, the final dataset for these analyses is 3285 subjects (1827 males and 1458 females) between ages 7 to 96 years old.

### 2.2. Pathology Review

Age at diagnosis, sex, and anatomic site of the melanoma were extracted from pathology reports and confirmed during patient interview. Histologic subtype and Breslow thickness were also extracted from pathology reports. The diagnostic slides underwent centralized pathology slide review for histopathologic characteristics [30,32,33,34], according to established criteria [35,36]. The pathology slide review included evaluation of histologic subtype, Breslow thickness, ulceration, mitoses, and TIL grade. The histologic subtype from the centralized review was chosen unless missing, in which case the subtype from the pathology report was utilized. Breslow thickness was obtained from both sources, and the measure corresponding to the deepest reading was chosen to represent the value of most biological relevance. Ulceration, mitoses, and TIL grade were only obtained from the centralized review, as these characteristics are less reliably documented in pathology reports. Ulceration and mitoses were recorded as present or absent [37]. TIL grade was scored as brisk, nonbrisk, or absent using a previously defined grading system [38,39,40]. Missing data resulted from a lack of access to the diagnostic slide or transection of the melanoma. Breslow thickness has less missing data than ulceration, mitoses, and TIL grade because these latter characteristics were only obtained from centralized review, whereas Breslow thickness was obtained from both the centralized review and the pathology report. The pathologists conducting the centralized review were blinded to genotype and survival.

### 2.3. Genotyping

SNPs were selected, as described [3], based on their association with melanoma in other studies and genotyped from buccal swab DNA using the MassArray iPLEX assay (Agena Bioscience, San Diego, CA, USA; previously known as Sequenom) with reported quality control measures [41]. The staff running assays were blinded to outcomes.

### 2.4. Survival

Information about deaths from melanoma or other causes was obtained for all participants from National Death Indexes, cancer registries, and municipal records. Patient follow-up for vital status was complete through 2008 for British Columbia, Canada, and Turin, Italy, and to the end of 2007 for all other centers.

### 2.5. Statistical Analysis

Breslow thickness was normalized using a log transformation. Linear regression models estimated the per allele mean changes in log of Breslow thickness and 95% confidence intervals (CIs) for each SNP. TIL grade was dichotomized as present (brisk or nonbrisk) or absent. Logistic regression models estimated the per allele odds ratios (ORs) and 95% CIs for presence versus absence of ulceration, mitoses, or TILs for each SNP. These models were all adjusted for baseline features (age at diagnosis, sex, and study center) and lesion status as first- or higher-order primary. We performed a principal component analysis of the 47 SNPs to detect potential population structure within our data, as described previously [42].

We next explored melanoma-specific survival. For these analyses, we limited the dataset to 2458 patients of self-reported European origin who entered the study with invasive first-order primary melanoma during the ascertainment period. Patients that entered the study with second- or higher-order primary melanoma during the ascertainment period were not included. For these patients, it would have been necessary to account for previous melanomas that occurred prior to the ascertainment period, which was not included in this investigation. Survival time was accumulated from the diagnosis date until the date of death due to melanoma, date of death due to any cause other than melanoma, or the end of follow-up (censored patients). The median follow-up time was 7.7 years. Cox proportional hazards regression analyses estimated the hazard ratios (HRs) and 95% CIs for the per allele association of each SNP with melanoma-specific survival adjusted for baseline features. In this analysis, for cases who developed a second primary melanoma, the occurrence of the second primary was included as a time-dependent covariate.

The false discovery threshold adjusted for multiple comparisons was computed using a resampling method that considers the linkage disequilibrium information among SNPs evaluated and is less conservative than the classical Bonferroni procedure [43,44]. All tests were two-sided. Data were analyzed using Stata/SE 16.1 (College Station, TX, USA).

## 3. Results

The demographic and tumor characteristics of the 3285 GEM participants of European origin with incident invasive primary melanoma included in these analyses are in Table 1. The median age was 58 years and 55.6% were male. Most melanomas (43.7%) were on the trunk with smaller proportions on the head or neck (17.2%), upper extremities (18.1%), and lower extremities (20.9%). The predominant subtype was superficial spreading melanoma (65.3%). The melanomas had a median thickness of 0.70 mm (interquartile range = 0.44–1.26 mm); 6.8% had ulceration present, 32.9% had mitoses present, and 62.2% had TILs (brisk or nonbrisk TIL grade) present. The locations, minor alleles, minor allele frequencies in GEM, and literature references for the 47 SNPs are in Table S1. The numbers of samples genotyped are in Table S2.

Passing the false discovery threshold (*p* = 0.0026) were associations of *IRF4* rs12203592 and *CCND1* rs1485993 with log of Breslow thickness, and association of *TERT* rs2242652 with presence of mitoses (Table 2). Adjusting for the top two principal components from our principal component analysis did not affect these associations (OR change 0–1%, results not shown). No SNPs passed false discovery for their association with presence of ulceration or TILs or melanoma-specific survival. Nominal associations (*p* < 0.05) with prognostic characteristics and melanoma-specific survival are in Table 2 and Table 3, respectively.

In addition to *IRF4* rs12203592*T passing false discovery for its association with increased log of Breslow thickness, *IRF4* rs12203592*T had nominal associations (*p* < 0.05) with presence of mitoses and worse melanoma-specific survival, as well as a borderline association (*p* = 0.07) with presence of ulceration.

In addition to *CCND1* rs1485993*T passing false discovery for its association with decreased log of Breslow thickness, *CCND1* rs1485993*T was borderline associated with absence of mitoses (*p* = 0.06). Also, *CCND1* rs11604821*G and rs11263498*T were each nominally associated with both decreased log of Breslow thickness and absence of mitoses. While *TERT* rs2242652 did not have any additional nominal associations, *TERT* rs2853676*A was nominally associated with absence of mitoses, and *TERT*; *CLPTM1L* rs401681*T was nominally associated with decreased log of Breslow thickness and absence of mitoses. *MX2* rs45430*G had nominal associations with decreased log of Breslow thickness and absence of mitoses, as well as better melanoma-specific survival.

We have previously reported, in separate and combined analyses of GEM and the Western Australia Melanoma Health Study (WAMHS), the associations of *IRF4* rs12203592, *CCND1* rs11263498, and *MX2* rs45430 with Breslow thickness [45] and *IRF4* rs12203592 with melanoma-specific survival among first-order primary melanoma patients [46].

## 4. Discussion

Our results indicate that many of these 47 melanoma-risk SNPs are not significantly associated with tumor prognostic characteristics or melanoma-specific survival when considering false discovery. Similarly, Mangantig et al., in a GWAS meta-analysis, found no significant associations with log of Breslow thickness for the *ARNT*, *PARP1*, *NID1*, *TERT*, *SLC45A2*, *MTAP*, *TYR*, *OCA2*, *HERC2*, *ASIP*, *PIGU*, or *PLA2G6* variants we studied [47]. Mangantig et al. also found no significant association with log of Breslow thickness for *CCND1* rs11263498 [47], while this SNP was nominally associated with log of Breslow thickness in GEM.

Consistent with GEM, Mangantig et al. found *IRF4* rs12203592*T was positively associated with increased log of Breslow thickness, although not reaching genome-wide significance [47]. Similarly consistent with GEM, Potrony et al. found that *IRF4* rs12203592*T increased the risk of dying from melanoma in patients from two European hospitals [48]. The *IRF4* rs12203592*T allele was the melanoma-risk allele in two US studies [22,23], while it was protective in a Spanish population [20] as well as a combined analysis of Australian, UK, and Swedish subjects [21]. Here, we report the overall positive associations of *IRF4* rs12203592*T with increased log of Breslow thickness, presence of mitoses, and worse melanoma-specific survival, along with a borderline association with presence of ulceration. *IRF4* is a transcription factor required for the maturation of B and T cells and for the differentiation of B lymphocytes into plasma cells [49]. In immune cells, the *IRF4* rs12203592*T allele increases *IRF4* expression, which upregulates telomerase activity by activating *TERT* transcription [50,51,52]. Furthermore, it has been suggested that increased *IRF4* expression in immune cells increases the ability of regulatory T cells to suppress T_H_2 responses [53], which may accelerate tumor growth. However, it has also been shown that *IRF4* overexpression in myeloid-derived suppressor cells induces a decreased suppressive effect on CD8+ T cell proliferation, resulting in less rapid tumor progression [54,55].

The melanoma-risk *CCND1* rs1485993*T allele [5] was positively associated with decreased Breslow thickness, passing false discovery, and borderline associated with absence of mitoses. The melanoma-risk alleles of other SNPs in the *CCND1* gene neighborhood (rs11604821*G and rs11263498*T) [5] were nominally associated with both decreased Breslow thickness and absence of mitoses. These results are plausible based on *CCND1*′s impact on cell proliferation [56]. *CCND1* is a cyclin that associates with CDK4 or CDK6 to inactivate the cell cycle inhibiting the function of the retinoblastoma protein (pRB), which promotes progression through the G_1_-S phase of the cell cycle [56,57]. It is interesting that the melanoma-risk alleles were associated with decreased Breslow thickness and absence of mitoses. This indicates that while these variants are related to increased melanoma susceptibility, they may also be associated with decreased tumor aggressivity. A recent meta-analysis investigating the associations of *CCND1* and cyclin protein D1 with melanoma prognostic factors found that upregulation of *CCND1*/cyclin D1 was associated with the presence of ulceration and mitoses, while the associations with Breslow thickness and survival conflicted across studies [58]. However, the associations of *CCND1* rs1485993*T, *CCND1* rs11604821*G, and *CCND1* rs11263498*T with *CCND1* expression remain unknown, and thus, we are unable to establish whether our results are consistent with the prior studies evaluating prognostic factors in the context of *CCND1* expression.

The melanoma-risk *TERT* rs2242652*T allele [24] was positively associated with absence of mitoses, passing false discovery. The melanoma-risk *TERT* rs2853676*A allele [24] was also nominally associated with absence of mitoses, and the melanoma-risk rs401681*T allele [5,24,59,60] was nominally associated with decreased log of Breslow thickness and absence of mitoses. These results are reasonable based on *TERT*’s regulation of telomerase activity [61]. Again, it is notable that the melanoma-risk alleles were associated with decreased Breslow thickness and/or absence of mitoses. Activating *TERT* promoter mutations result in increased gene expression and have been associated with increased Breslow thickness and the presence of ulceration and mitoses in melanoma patients [62,63,64,65,66]. Other studies have not found associations between *TERT* promoter mutations and Breslow thickness, ulceration, or mitotic rate [67,68,69]. However, similarly to *CCND1*, the associations of our genotypes with *TERT* expression remain unknown, and thus, we are unable to establish whether our results are consistent with these prior studies.

Also noteworthy are the results that the melanoma-risk *MX2* rs45430*A allele [5] was positively associated with increased Breslow thickness, presence of mitoses, and worse melanoma-specific survival in GEM. *MX2* is a dynamin-like GTPase that is an interferon-induced inhibitor of HIV-1 and other primate lentiviruses [70]. Impairing *MX2* function also leads to a delay in progression through the G_1_-S phase of the cell cycle [71]. Although Mangantig et al. found no association of *MX2* rs45430 with Breslow thickness [47], other studies indicate *MX2* may influence melanoma progression [72,73]. *MX2* rs45430 is in linkage disequilibrium with *MX2* rs398206 (D’ = 0.98 in the CEU population), and *MX2* rs45430*A is strongly positively correlated with *MX2* rs398206*A [74]. Choi et al. identified *MX2* rs398206 as a functional intronic variant that mediates Yingyang-1 (YY1) binding to increase *MX2* levels, with *MX2* rs398206*A driving significantly higher luciferase expression compared to the C allele [72]. They further found that melanocyte-specific expression of human *MX2* in a zebrafish model accelerated melanoma formation in a *BRAF*V600E background. Juraleviciute et al. found that primary melanomas homozygous for *MX2* rs45430*A had higher *MX2* expression [73]. Interestingly, these authors found the effects of *MX2* expression on melanoma proliferation were context-dependent, with high expression in primary melanoma cell lines and melanocytes suppressing tumorigenesis, while downregulation in a subset of melanoma cell lines reduced proliferation. These differential effects in melanoma subsets may obscure associations in epidemiologic studies. Juraleviciute et al. also reported that *MX2* expression was significantly higher in tumors with TILs compared to tumors that had no TILs [73]. Here, we found no significant association with TILs for *MX2* rs45430.

Our study’s strengths are its international population-based design, large sample size, standardized pathology review, melanoma-specific survival, and comparatively long follow-up period ending before approvals of new systemic agents, checkpoint inhibitors, and targeted therapies that alter the natural course of the disease and improve overall survival [75]. Future studies examining melanoma-specific survival will likely be confounded by these new therapies. A limitation could be insufficient power to detect associations of SNPs with lower minor allele frequencies (e.g., *SLC45A2* rs16891982, MAF 0.017). Another limitation is that our study only included participants with cutaneous melanoma, not mucosal [76,77] or uveal melanomas [78,79,80], which seemingly have different genetic landscapes.

## 5. Conclusions

Our findings indicate that few melanoma-risk variants are associated with tumor prognostic characteristics (Breslow thickness, presence of ulceration, presence of mitoses, or presence of TILs) or survival. However, further research investigating the associations of *IRF4* rs12203592, *CCND1* rs1485993, *TERT* rs2242652, and *MX2* rs45430 with underlying biologic pathways related to tumor progression is warranted. Future studies of larger datasets that include subset analyses may help elucidate the relationship of melanoma-risk variants with tumor characteristics and survival.

## Figures and Tables

**Table 1:** Characteristics of patients with incident invasive cutaneous melanoma in the GEM study (*n* = 3285) ^1^.

Characteristic	No. (%)
Median age at most recent diagnosis (IQR), years	58 (46–70)
Sex	
Male	1827 (55.6)
Female	1458 (44.4)
Lesion status	
First-order primary melanoma	2458 (74.8)
Higher-order primary melanoma	827 (25.2)
Anatomic site	
Head/neck	565 (17.2)
Trunk	1437 (43.7)
Upper extremities	595 (18.1)
Lower extremities	688 (20.9)
Histologic subtype	
Superficial spreading	2144 (65.3)
Nodular	275 (8.4)
Lentigo maligna	377 (11.5)
Unclassified/other ^2^	489 (14.9)
Breslow thickness, mm	
Median (IQR)	0.70 (0.44–1.26)
0.01–1.00	2195 (66.8)
1.01–2.00	592 (18.0)
2.01–4.00	276 (8.4)
>4.00	144 (4.4)
Missing	78 (2.4)
Ulceration	
Absent	2392 (72.8)
Present	225 (6.8)
Missing	668 (20.3)
Mitoses	
Absent	1544 (47.0)
Present	1081 (32.9)
Missing	660 (20.1)
Tumor-infiltrating lymphocyte (TIL) grade	
Absent	567 (17.3)
Nonbrisk	1658 (50.5)
Brisk	385 (11.7)
Missing	675 (20.5)

Abbreviations: GEM, Genes, Environment and Melanoma; No., number; IQR, interquartile range. ^1^ Limited to individuals of European origin with incident invasive first- or higher-order primary melanoma. Percentages may not sum to 100 because of rounding of decimals. ^2^ Other includes acral lentiginous, spindle cell, nevoid, and Spitzoid melanomas.

**Table 2:** Associations of melanoma-risk SNPs with primary melanoma tumor prognostic characteristics among patients in the GEM study ^1^.

			Tumor Prognostic Characteristics								
			Breslow Thickness (*n* = 3207)			Present vs. Absent Ulceration (*n* = 2617)		Present vs. Absent Mitoses (*n* = 2625)		Nonbrisk/Brisk vs. Absent TIL grade (*n* = 2610)	
Gene Neighborhood	SNP	a/A	Per allele mean change in log of Breslow thickness (95% CI) ^2^	Per allele change in Breslow thickness, % ^3^	*p*	Per allele OR (95% CI) ^4^	*p*	Per allele OR (95% CI) ^4^	*p*	Per allele OR (95% CI) ^4^	*p*
*ARNT*	rs7412746	C/T	0.02 (−0.02–0.06)	2.15	0.30	1.11 (0.91–1.35)	0.32	**1.12 (1.00–1.25)**	**0.04**	0.90 (0.79–1.03)	0.12
*PARP1*	rs3219090	A/G	0.004 (−0.04–0.05)	0.44	0.85	0.96 (0.77–1.19)	0.70	0.96 (0.85–1.08)	0.48	1.07 (0.93–1.25)	0.34
*PARP1*	rs2695238	C/G	0.01 (−0.03–0.06)	1.11	0.62	0.96 (0.78–1.19)	0.73	0.97 (0.86–1.09)	0.60	1.03 (0.89–1.19)	0.69
*NID1*	rs3768080	G/A	−0.03 (−0.07–0.006)	−3.35	0.10	0.83 (0.68–1.01)	0.06	0.91 (0.81–1.02)	0.10	0.95 (0.83–1.08)	0.42
*NID1*	rs10754833	C/T	−0.03 (−0.07–0.006)	−3.33	0.10	0.83 (0.68–1.01)	0.06	0.90 (0.81–1.01)	0.08	0.94 (0.83–1.08)	0.40
*CASP8*	rs6735656 ^a^	G/T	−0.02 (−0.06–0.03)	−1.69	0.47	0.95 (0.76–1.19)	0.65	0.96 (0.85–1.09)	0.53	0.97 (0.83–1.13)	0.67
*CASP8*	rs13016963	A/G	−0.01 (−0.05–0.03)	−1.03	0.62	1.02 (0.84–1.25)	0.81	0.91 (0.81–1.02)	0.10	1.01 (0.88–1.16)	0.90
*TERT*	rs2242652	T/C	−0.04 (−0.09–0.02)	−3.56	0.17	0.99 (0.77–1.27)	0.92	**0.80 (0.69–0.92)**	**0.002**	1.05 (0.89–1.25)	0.55
*TERT*	rs2853676	A/G	−0.02 (−0.06–0.03)	−1.69	0.45	0.96 (0.77–1.19)	0.69	**0.87 (0.77–0.98)**	**0.02**	0.95 (0.82–1.10)	0.48
*TERT*	rs13356727	G/A	−0.03 (−0.07–0.007)	−3.29	0.11	0.92 (0.76–1.12)	0.41	0.91 (0.82–1.02)	0.10	0.92 (0.81–1.06)	0.24
*TERT*;* CLPTM1L*	rs4975616	G/A	−0.03 (−0.07–0.01)	−2.96	0.16	1.03 (0.84–1.27)	0.79	0.93 (0.83–1.04)	0.22	0.93 (0.81–1.07)	0.33
*TERT*;* CLPTM1L*	rs401681	T/C	**−0.05 (−0.09 to −0.007)**	**−4.64**	**0.02**	0.94 (0.77–1.14)	0.51	**0.88 (0.79–0.99)**	**0.03**	0.98 (0.86–1.12)	0.80
*SLC45A2*	rs16891982	C/G	0.03 (−0.13–0.19)	2.93	0.72	1.12 (0.55–2.30)	0.76	0.73 (0.47–1.14)	0.16	**0.63 (0.40–0.99)**	**0.05**
*SLC45A2*	rs35391	T/C	0.08 (−0.12–0.28)	8.57	0.41	1.52 (0.66–3.52)	0.33	0.91 (0.53–1.57)	0.73	0.71 (0.39–1.29)	0.26
*SLC45A2*	rs26722	T/C	0.04 (−0.17–0.25)	3.76	0.73	1.28 (0.49–3.32)	0.61	0.86 (0.48-–1.52)	0.60	0.84 (0.44–1.62)	0.61
*SLC45A2*	rs13289	G/C	0.03 (−0.01–0.07)	2.87	0.19	1.11 (0.90–1.35)	0.33	1.09 (0.97–1.22)	0.14	**0.82 (0.72–0.94)**	**0.005**
*IRF4*	rs12203592	T/C	**0.08 (0.03–0.13)**	**8.14**	**0.002**	1.23 (0.99–1.54)	0.07	**1.17 (1.02–1.33)**	**0.02**	0.92 (0.79–1.08)	0.31
*IRF4*	rs872071	A/G	0.008 (−0.03–0.05)	0.76	0.71	0.94 (0.77–1.14)	0.54	1.05 (0.94–1.17)	0.42	1.00 (0.87–1.14)	0.97
*TYRP1*	rs1408799	T/C	0.008 (−0.04–0.05)	0.85	0.71	1.19 (0.97–1.47)	0.09	1.09 (0.97–1.23)	0.15	1.05 (0.90–1.21)	0.54
*TYRP1*	rs2733832	C/T	0.02 (−0.02–0.06)	1.77	0.41	1.05 (0.86–1.28)	0.65	1.08 (0.97–1.22)	0.17	1.05 (0.91–1.20)	0.53
*MTAP*	rs2218220	T/C	0.005 (−0.04–0.04)	0.48	0.82	0.97 (0.80–1.18)	0.79	1.01 (0.90–1.12)	0.92	**1.15 (1.00–1.31)**	**0.04**
*MTAP*	rs1335510	G/T	0.003 (−0.04–0.04)	0.26	0.90	0.89 (0.73–1.09)	0.28	1.00 (0.89–1.12)	0.95	**1.18 (1.03–1.35)**	**0.02**
*MTAP*	rs7023329	G/A	0.01 (−0.03–0.05)	1.22	0.55	0.98 (0.80–1.19)	0.82	0.98 (0.88–1.10)	0.74	1.10 (0.96–1.26)	0.16
*MTAP*	rs10811629	G/A	0.006 (−0.03–0.05)	0.61	0.77	0.97 (0.80–1.19)	0.79	1.03 (0.92–1.15)	0.60	1.12 (0.98–1.28)	0.10
*CCND1*	rs11604821	G/A	**−0.06 (−0.11 to −0.02)**	**−6.06**	**0.004**	0.98 (0.79–1.21)	0.84	**0.88 (0.78–0.99)**	**0.03**	0.98 (0.85–1.12)	0.73
*CCND1*	rs1485993	T/C	**−0.07 (−0.11 to −0.03)**	**−6.77**	**0.001**	1.01 (0.83–1.24)	0.89	0.89 (0.79–1.00)	0.06	1.03 (0.89–1.18)	0.70
*CCND1*	rs11263498	T/C	**−0.06 (−0.10 to −0.02)**	**−5.78**	**0.006**	0.96 (0.78–1.19)	0.73	**0.89 (0.79–1.00)**	**0.04**	1.01 (0.88–1.16)	0.89
*TYR*	rs1042602	A/C	0.008 (−0.03–0.05)	0.75	0.73	1.07 (0.87–1.32)	0.50	0.94 (0.84–1.06)	0.31	1.08 (0.94–1.25)	0.27
*TYR*	rs10765198	C/T	0.01 (−0.03–0.06)	1.40	0.52	0.95 (0.77–1.17)	0.62	1.12 (1.00–1.26)	0.05	0.98 (0.85–1.12)	0.72
*TYR*	rs1847142	A/G	0.01 (−0.03–0.05)	1.30	0.54	1.02 (0.84–1.25)	0.82	1.08 (0.97–1.21)	0.17	0.94 (0.82–1.08)	0.41
*TYR*	rs10830253	G/T	0.01 (−0.03–0.05)	0.98	0.65	1.01 (0.82–1.24)	0.92	1.08 (0.96–1.21)	0.19	0.93 (0.81–1.07)	0.29
*ATM*	rs12278954 ^b^	A/C	0.02 (−0.04–0.07)	1.59	0.59	1.04 (0.79–1.37)	0.76	0.92 (0.79–1.08)	0.31	0.99 (0.82–1.20)	0.94
*OCA2*	rs1800407	A/G	0.004 (−0.07–0.07)	0.41	0.91	0.99 (0.71–1.40)	0.97	0.92 (0.76–1.12)	0.42	0.88 (0.71–1.11)	0.28
*OCA2*	rs1800401	T/C	−0.02 (−0.12–0.07)	−2.37	0.61	1.06 (0.68–1.67)	0.80	1.08 (0.83–1.40)	0.56	1.23 (0.89–1.70)	0.22
*HERC2*	rs1129038	G/A	0.02 (−0.03–0.07)	2.29	0.37	1.15 (0.91–1.45)	0.26	0.97 (0.85–1.12)	0.72	0.93 (0.79–1.10)	0.39
*HERC2*	rs12913832	A/G	0.02 (−0.03–0.07)	2.03	0.42	1.12 (0.89–1.42)	0.34	0.96 (0.84–1.10)	0.60	0.95 (0.80–1.11)	0.51
*ASIP*	rs17305657	C/T	−0.03 (−0.10–0.03)	−3.36	0.31	0.77 (0.54–1.10)	0.15	0.87 (0.72–1.05)	0.14	1.11 (0.89–1.39)	0.35
*ASIP*	rs4911414	T/G	−0.02 (−0.06–0.02)	−2.07	0.33	1.02 (0.83–1.25)	0.88	0.89 (0.80–1.01)	0.06	1.05 (0.92–1.21)	0.47
*PIGU*	rs910873	A/G	−0.02 (−0.09–0.04)	−2.42	0.44	0.77 (0.56–1.07)	0.12	0.86 (0.72–1.03)	0.09	1.10 (0.89–1.36)	0.37
*PIGU*	rs17305573	C/T	−0.01 (−0.08–0.05)	−1.26	0.71	0.74 (0.51–1.06)	0.10	0.86 (0.71–1.04)	0.11	1.05 (0.84–1.32)	0.65
*NCOA6*	rs4911442	G/A	−0.01 (−0.07–0.04)	−1.28	0.65	0.83 (0.62–1.11)	0.22	0.88 (0.75–1.03)	0.10	1.11 (0.92–1.35)	0.27
*MYH7B*	rs1885120	C/G	−0.04 (−0.11–0.02)	−4.31	0.18	**0.64 (0.44–0.92)**	**0.02**	0.85 (0.71–1.02)	0.08	1.12 (0.90–1.4)	0.30
*LOC647979*	rs1204552	A/T	−0.02 (−0.09–0.05)	−1.77	0.63	0.93 (0.66–1.33)	0.71	0.91 (0.75–1.11)	0.35	1.05 (0.83–1.33)	0.68
*MX2*	rs45430	G/A	**−0.06 (−0.11 to −0.02)**	**−6.14**	**0.004**	0.90 (0.73–1.11)	0.34	**0.87 (0.77–0.97)**	**0.02**	1.12 (0.97–1.29)	0.13
*PLA2G6*	rs6001027	G/A	0.01 (−0.03–0.06)	1.35	0.54	0.87 (0.70–1.09)	0.23	0.93 (0.83–1.05)	0.25	0.94 (0.81–1.08)	0.39
*PLA2G6*	rs132985	T/C	0.01 (−0.03–0.05)	1.21	0.56	0.90 (0.74–1.10)	0.30	0.99 (0.89–1.11)	0.87	0.93 (0.82–1.07)	0.32
*PLA2G6*	rs738322	G/A	0.007 (−0.03–0.05)	0.75	0.72	0.94 (0.77–1.14)	0.51	1.00 (0.89–1.12)	1.00	0.93 (0.82–1.07)	0.31

Abbreviations: SNP, single nucleotide polymorphism; GEM, Genes, Environment and Melanoma; TIL, tumor-infiltrating lymphocyte; Chr, chromosome; a, minor allele; A, major allele; CI, confidence interval; OR, odds ratio. Bold type indicates *p* values ≤ 0.05 (two-sided). ^1^ Limited to 3285 to individuals of European origin with incident invasive first- or higher-order primary melanoma who had their melanoma scored for the histopathologic variable of interest (i.e., Breslow thickness, ulceration, mitoses, or TIL grade). ^2^ Adjusted for baseline features (age at diagnosis, sex, and study center) and status as first- or higher-order primary. The mean changes and 95% CIs per minor allele are provided. ^3^ As the outcome (Breslow thickness) was log-transformed, the values here are presented as 100 × (e^estimated beta coefficient^ − 1), which may be interpreted as the percentage change in the estimated mean of Breslow thickness per minor allele. ^4^ Adjusted for baseline features and status as first- or higher-order primary. The ORs and 95% CIs per minor allele are provided. ^a^ rs6735656 is a proxy for rs10931936 (r^2^ = 0.965). ^b^ rs12278954 is a proxy for rs1801516 (r^2^ =1.00).

**Table 3:** Associations of melanoma-risk SNPs with melanoma-specific survival among patients in the GEM study ^1^.

			Total	Censored	Death as a Result of Melanoma	Melanoma-Specific Survival	
Gene Neighborhood	SNP	a/A	No.	No.	No.	Per allele HR (95% CI) ^2^	*p*
*ARNT*	rs7412746	C/T	2420	2262	158	1.02 (0.82–1.28)	0.84
*PARP1*	rs3219090	A/G	2387	2232	155	1.18 (0.94–1.50)	0.16
*PARP1*	rs2695238	C/G	2428	2267	161	1.07 (0.85–1.35)	0.58
*NID1*	rs3768080	G/A	2409	2251	158	0.82 (0.66–1.02)	0.08
*NID1*	rs10754833	C/T	2419	2260	159	0.83 (0.66–1.03)	0.09
*CASP8*	rs6735656 ^a^	G/T	2400	2244	156	0.94 (0.73–1.21)	0.64
*CASP8*	rs13016963	A/G	2423	2264	159	0.93 (0.75–1.17)	0.55
*TERT*	rs2242652	T/C	2305	2153	152	0.96 (0.73–1.28)	0.80
*TERT*	rs2853676	A/G	2420	2259	161	0.96 (0.76–1.22)	0.73
*TERT*	rs13356727	G/A	2439	2279	160	0.94 (0.75–1.17)	0.59
*TERT*;* CLPTM1L*	rs4975616	G/A	2343	2193	150	0.94 (0.75–1.19)	0.61
*TERT*;* CLPTM1L*	rs401681	T/C	2408	2249	159	0.97 (0.77–1.21)	0.76
*SLC45A2*	rs16891982	C/G	2425	2265	160	1.29 (0.65–2.57)	0.46
*SLC45A2*	rs35391	T/C	2411	2254	157	0.75 (0.25–2.31)	0.62
*SLC45A2*	rs26722	T/C	2397	2239	158	1.36 (0.56–3.30)	0.49
*SLC45A2*	rs13289	G/C	2413	2252	161	0.82 (0.65–1.04)	0.10
*IRF4*	rs12203592	T/C	2425	2265	160	**1.28 (1.00–1.65)**	**0.05**
*IRF4*	rs872071	A/G	2406	2247	159	0.95 (0.76–1.18)	0.63
*TYRP1*	rs1408799	T/C	2401	2242	159	1.17 (0.93–1.46)	0.18
*TYRP1*	rs2733832	C/T	2405	2248	157	1.23 (0.98–1.53)	0.07
*MTAP*	rs2218220	T/C	2419	2258	161	1.05 (0.84–1.30)	0.68
*MTAP*	rs1335510	G/T	2404	2249	155	0.98 (0.78–1.23)	0.87
*MTAP*	rs7023329	G/A	2401	2244	157	1.05 (0.84–1.30)	0.69
*MTAP*	rs10811629	G/A	2414	2255	159	1.00 (0.80–1.25)	1.00
*CCND1*	rs11604821	G/A	2427	2269	158	1.02 (0.81–1.29)	0.86
*CCND1*	rs1485993	T/C	2410	2250	160	1.13 (0.90–1.42)	0.28
*CCND1*	rs11263498	T/C	2421	2263	158	1.12 (0.89–1.40)	0.35
*TYR*	rs1042602	A/C	2429	2270	159	0.90 (0.71–1.14)	0.38
*TYR*	rs10765198	C/T	2428	2270	158	0.90 (0.71–1.14)	0.37
*TYR*	rs1847142	A/G	2424	2264	160	0.91 (0.73–1.15)	0.45
*TYR*	rs10830253	G/T	2403	2247	156	0.92 (0.73–1.16)	0.48
*ATM*	rs12278954 ^b^	A/C	2429	2268	161	**1.37 (1.04–1.80)**	**0.03**
*OCA2*	rs1800407	A/G	2429	2270	159	**1.45 (1.02–2.04)**	**0.04**
*OCA2*	rs1800401	T/C	2434	2273	161	0.65 (0.34–1.21)	0.17
*HERC2*	rs1129038	G/A	2409	2252	157	**1.38 (1.07–1.77)**	**0.01**
*HERC2*	rs12913832	A/G	2429	2268	161	**1.38 (1.08–1.76)**	**0.01**
*ASIP*	rs17305657	C/T	2417	2257	160	0.98 (0.67–1.43)	0.92
*ASIP*	rs4911414	T/G	2426	2265	161	0.83 (0.66–1.05)	0.12
*PIGU*	rs910873	A/G	2431	2271	160	0.99 (0.70–1.41)	0.96
*PIGU*	rs17305573	C/T	2143	2003	140	1.00 (0.68–1.47)	0.99
*NCOA6*	rs4911442	G/A	2399	2241	158	1.00 (0.73–1.38)	0.98
*MYH7B*	rs1885120	C/G	2417	2259	158	0.95 (0.65–1.38)	0.79
*LOC647979*	rs1204552	A/T	2356	2202	154	1.09 (0.74–1.62)	0.67
*MX2*	rs45430	G/A	2421	2259	162	**0.79 (0.62–0.99)**	**0.05**
*PLA2G6*	rs6001027	G/A	2281	2133	148	1.17 (0.92–1.48)	0.20
*PLA2G6*	rs132985	T/C	2422	2263	159	1.07 (0.85–1.33)	0.57
*PLA2G6*	rs738322	G/A	2412	2254	158	1.14 (0.91–1.42)	0.25

Abbreviations: SNP, single nucleotide polymorphism; GEM, Genes, Environment and Melanoma; Chr, chromosome; a, minor allele; A, major allele; CI, confidence interval; HR; hazard ratio. Bold type indicates *p* values ≤ 0.05 (two-sided). ^1^ Limited to 2458 individuals of European origin with incident invasive first-order primary melanoma. ^2^ Adjusted for baseline features (age at diagnosis, sex, and study center) and a time-dependent covariate. The HRs and 95% CIs per minor allele are provided. ^a^ rs6735656 is a proxy for rs10931936 (r^2^ = 0.965). ^b^ rs12278954 is a proxy for rs1801516 (r^2^ = 1.00).

## Data Availability

Data can be requested from Nancy E. Thomas or Marianne Berwick after review by the GEM Steering Committee.

## Supplementary Materials

The following are available online at https://www.mdpi.com/article/10.3390/curroncol28060401/s1, Table S1: Genotype locations, minor/major alleles, minor allele frequencies, numbers of samples genotyped in the GEM study, and references for the association of the genotypes with melanoma; Table S2: Number of GEM participants successfully genotyped for each SNP and distribution of these participants by prognostic characteristics of primary melanoma tumor.
