# Supplementary material for: Association of Melanoma-Risk Variants with Primary Melanoma Tumor Prognostic Characteristics and Melanoma-Specific Survival in the GEM Study
Source: Curr Oncol. 2021 Nov 16;28(6):4756–71. doi: 10.3390/curroncol28060401 (PMC8628692; doi:10.3390/curroncol28060401)
Supplement: Supplementary file 1 [file curroncol-28-00401-s001.zip › curroncol-1358237-supplementary.pdf]

Supplementary Table S1. Genotype locations, minor/major alleles, minor allele frequencies, numbers of samples genotyped in the GEM study, and references for the association of the genotypes with melanoma[1]

Chromosome	Gene neighborhood	SNP	Location[2]	a/A	MAF	No. genotyped	No. higher-order primary melanoma aa/aA/AA	No. first-order primary melanoma aa/aA/AA	References	RegulomeDB Rank / Score[3]
1	*ARNT*	rs7412746	150860471	C/T	0.44	3236	151/382/282	491/1150/780	1, 2, 3	6 / 0.16
1	*PARP1*	rs3219090	226564691	A/G	0.32	3198	71/356/383	243/1071/1074	3, 4	7 / 0.18
1	*PARP1*	rs2695238	226604519	C/G	0.31	3250	72/357/392	237/1052/1140	3	5 / 0.62
1	*NID1*	rs3768080	236179869	G/A	0.48	3228	178/415/225	576/1161/673	5	7 / 0.18
1	*NID1*	rs10754833	236184931	C/T	0.48	3236	184/409/223	591/1162/667	5	3a / 0.55
2	*CASP8*	rs6735656[a]	202124502	G/T	0.27	3213	61/329/422	171/925/1305	6	1f / 0.55
2	*CASP8*	rs13016963	202162811	A/G	0.38	3244	133/374/313	364/1113/947	6	5 / 0.0
5	*TERT*	rs2242652	1280028	T/C	0.21	3103	41/258/498	94/754/1458	7	2b / 0.80
5	*TERT*	rs2853676	1288547	A/G	0.29	3244	76/329/418	229/944/1248	7	5 / 0.13
5	*TERT*	rs13356727	1312457	G/A	0.46	3262	188/421/213	496/1193/751	8	4 / 0.61
5	*TERT;CLPTM1L*	rs4975616	1315660	G/A	0.48	3145	189/424/188	502/1193/649	8	4 / 0.61
5	*TERT;CLPTM1L*	rs401681	1322087	T/C	0.48	3225	215/415/186	539/1184/686	6, 7, 8, 9	5 / 0.13
5	*SLC45A2*	rs16891982	33951693	C/G	0.02	3244	0/11/807	4/90/2332	1, 6, 10, 11, 12	4 / 0.61
5	*SLC45A2*	rs35391	33955673	T/C	0.01	3222	0/4/806	2/59/2351	1, 11	7 / 0.18
5	*SLC45A2*	rs26722	33963870	T/C	0.01	3210	0/7/805	1/56/2341	12	6 / 0.0
5	*SLC45A2*	rs13289	33986409	G/C	0.37	3229	110/386/319	328/1130/956	13	3a / 0.98
6	*IRF4*	rs12203592	396321	T/C	0.23	3245	67/297/455	126/801/1499	4, 14, 15, 16	2b / 0.62
6	*IRF4*	rs872071	411064	A/G	0.45	3220	157/408/248	542/1114/751	6, 14	4 / 0.61
9	*TYRP1*	rs1408799	12672097	T/C	0.30	3218	62/334/420	250/978/1174	10, 11	5 / 1.0
9	*TYRP1*	rs2733832	12704725	C/T	0.40	3218	103/375/334	408/1139/859	11	3a / 0.55
9	*MTAP*	rs2218220	21756089	T/C	0.47	3241	191/396/234	563/1167/690	1, 10, 17	5 / 0.42
9	*MTAP*	rs1335510	21757803	G/T	0.37	3220	121/358/336	376/1100/929	1, 10, 17	1f / 0.55
9	*MTAP*	rs7023329	21816528	G/A	0.46	3216	176/393/245	552/1125/725	1, 10, 17, 18, 19	5 / 0.59
9	*MTAP*	rs10811629	21840298	G/A	0.39	3234	129/370/320	410/1125/880	17, 20	5 / 0.25
11	*CCND1*	rs11604821	69352137	G/A	0.36	3246	98/381/339	300/1130/998	6	5 / 0.13
11	*CCND1*	rs1485993	69362414	T/C	0.37	3228	113/379/325	333/1131/947	6	5 / 0.48
11	*CCND1*	rs11263498	69382767	T/C	0.37	3235	106/382/325	325/1147/950	6	4 / 0.61
11	*TYR*	rs1042602	88911696	A/C	0.35	3252	96/376/350	331/1103/996	1	5 / 0.13
11	*TYR*	rs10765198	88969774	C/T	0.34	3252	128/371/324	283/1034/1112	11	7 / 0.18
11	*TYR*	rs1847142	89021574	A/G	0.37	3246	140/385/296	326/1068/1031	1	7 / 0.18
11	*TYR*	rs10830253	89028043	G/T	0.37	3222	142/381/295	325/1052/1027	1	5 / 0.13
11	*ATM*	rs12278954[b]	108328952	A/C	0.14	3251	22/170/629	62/573/1795	6	5 / 0.13
15	*OCA2*	rs1800407	28230318	A/G	0.09	3249	14/151/654	20/381/2029	11	5 / 0.13
15	*OCA2*	rs1800401	28260053	T/C	0.05	3259	2/81/741	8/215/2212	21	4 / 0.61
15	*HERC2*	rs1129038	28356859	G/A	0.23	3225	42/271/502	139/847/1424	1, 2	5 / 0.13
15	*HERC2*	rs12913832	28365618	A/G	0.23	3250	42/274/504	143/849/1438	1, 2	5 / 0.63
20	*ASIP*	rs17305657	31806588	C/T	0.11	3237	13/175/631	25/434/1959	6, 22	6 / 0.27
20	*ASIP*	rs4911414	32729444	T/G	0.38	3248	126/417/278	331/1117/979	6	5 / 0.59
20	*PIGU*	rs910873	33171772	A/G	0.12	3256	16/203/605	27/514/1891	1, 10, 22, 23	1f / 0.55
20	*PIGU*	rs17305573	33180152	C/T	0.12	2904	14/178/568	24/426/1694	1, 18	5 / 0.80
20	*NCOA6*	rs4911442	33355046	G/A	0.16	3213	29/244/540	54/605/1741	1, 2, 6, 18, 22	7 / 0.18
20	*MYH7B*	rs1885120	33576989	C/G	0.11	3236	14/179/625	23/468/1927	1, 10, 23	1f / 0.55
20	*LOC647979*	rs1204552	34638903	A/T	0.09	3158	8/155/638	15/381/1961	10	4 / 0.61
21	*MX2*	rs45430	42746081	G/A	0.36	3240	95/375/348	333/1139/950	6	2b / 0.39
22	*PLA2G6*	rs6001027	38545619	G/A	0.34	3061	102/350/327	270/1003/1009	1, 17, 18	4 / 0.61
22	*PLA2G6*	rs132985	238563471	T/C	0.45	3242	161/401/257	489/1200/734	2, 17	5 / 0.13
22	*PLA2G6*	rs738322	238569006	G/A	0.45	3230	163/400/254	498/1198/717	17	4 / 0.61

Abbreviations: GEM, Genes, Environment and Melanoma; SNP, single nucleotide polymorphism; a, minor allele; A, major allele; MAF, minor allele frequency for all participants; No., number.

[1]Limited to individuals of European origin with incident invasive first- or higher-order primary melanoma.

[2]Location is determined using hg19.

[3]Accessed from RegulomeDB (https://regulomedb.org/).
[a]rs6735656 is a proxy for rs10931936 (r^2=0.965).
[b]rs12278954 is a proxy for rs1801516 (r^2=1.00).

REFERENCES

1. Amos CI, Wang LE, Lee JE, et al. Genome-wide association study identifies novel loci predisposing to cutaneous melanoma. Hum Mol Genet. Dec 15 2011;20(24):5012-23. doi:10.1093/hmg/ddr415
2. Fang S, Han J, Zhang M, et al. Joint effect of multiple common SNPs predicts melanoma susceptibility. PLoS One. 2013;8(12):e85642. doi:10.1371/journal.pone.0085642
3. Macgregor S, Montgomery GW, Liu JZ, et al. Genome-wide association study identifies a new melanoma susceptibility locus at 1q21.3. Nat Genet. Oct 9 2011;43(11):1114-8. doi:10.1038/ng.958
4. Peña-Chilet M, Blanquer-Maceiras M, Ibarrola-Villava M, et al. Genetic variants in PARP1 (rs3219090) and IRF4 (rs12203592) genes associated with melanoma susceptibility in a Spanish population. BMC Cancer. Mar 27 2013;13:160. doi:10.1186/1471-2407-13-160
5. Nan H, Xu M, Zhang J, et al. Genome-wide association study identifies nidogen 1 (NID1) as a susceptibility locus to cutaneous nevi and melanoma risk. Hum Mol Genet. Jul 1 2011;20(13):2673-9. doi:10.1093/hmg/ddr154
6. Barrett JH, Iles MM, Harland M, et al. Genome-wide association study identifies three new melanoma susceptibility loci. Nat Genet. Oct 9 2011;43(11):1108-13. doi:10.1038/ng.959
7. Nan H, Qureshi AA, Prescott J, De Vivo I, Han J. Genetic variants in telomere-maintaining genes and skin cancer risk. Hum Genet. Mar 2011;129(3):247-53. doi:10.1007/s00439-010-0921-5
8. Law MH, Montgomery GW, Brown KM, et al. Meta-analysis combining new and existing data sets confirms that the TERT-CLPTM1L locus influences melanoma risk. J Invest Dermatol. Feb 2012;132(2):485-7. doi:10.1038/jid.2011.322
9. Rafnar T, Sulem P, Stacey SN, et al. Sequence variants at the TERT-CLPTM1L locus associate with many cancer types. Nat Genet. Feb 2009;41(2):221-7. doi:10.1038/ng.296
10. Chatzinasiou F, Lill CM, Kypreou K, et al. Comprehensive field synopsis and systematic meta-analyses of genetic association studies in cutaneous melanoma. J Natl Cancer Inst. Aug 17 2011;103(16):1227-35. doi:10.1093/jnci/djr219
11. Duffy DL, Zhao ZZ, Sturm RA, Hayward NK, Martin NG, Montgomery GW. Multiple pigmentation gene polymorphisms account for a substantial proportion of risk of cutaneous malignant melanoma. J Invest Dermatol. Feb 2010;130(2):520-8. doi:10.1038/jid.2009.258
12. Guedj M, Bourillon A, Combadières C, et al. Variants of the MATP/SLC45A2 gene are protective for melanoma in the French population. Hum Mutat. Sep 2008;29(9):1154-60. doi:10.1002/humu.20823
13. Nan H, Kraft P, Hunter DJ, Han J. Genetic variants in pigmentation genes, pigmentary phenotypes, and risk of skin cancer in Caucasians. Int J Cancer. Aug 15 2009;125(4):909-17. doi:10.1002/ijc.24327
14. Duffy DL, Iles MM, Glass D, et al. IRF4 variants have age-specific effects on nevus count and predispose to melanoma. Am J Hum Genet. Jul 9 2010;87(1):6-16. doi:10.1016/j.ajhg.2010.05.017
15. Han J, Qureshi AA, Nan H, et al. A germline variant in the interferon regulatory factor 4 gene as a novel skin cancer risk locus. Cancer Res. Mar 1 2011;71(5):1533-9. doi:10.1158/0008-5472.Can-10-1818
16. Zhang M, Song F, Liang L, et al. Genome-wide association studies identify several new loci associated with pigmentation traits and skin cancer risk in European Americans. Hum Mol Genet. Jul 15 2013;22(14):2948-59. doi:10.1093/hmg/ddt142
17. Falchi M, Bataille V, Hayward NK, et al. Genome-wide association study identifies variants at 9p21 and 22q13 associated with development of cutaneous nevi. Nat Genet. Aug 2009;41(8):915-9. doi:10.1038/ng.410
18. Bishop DT, Demenais F, Iles MM, et al. Genome-wide association study identifies three loci associated with melanoma risk. Nat Genet. Aug 2009;41(8):920-5. doi:10.1038/ng.411
19. Newton-Bishop JA, Chang YM, Iles MM, et al. Melanocytic nevi, nevus genes, and melanoma risk in a large case-control study in the United Kingdom. Cancer Epidemiol Biomarkers Prev. Aug 2010;19(8):2043-54. doi:10.1158/1055-9965.Epi-10-0233
20. Maccioni L, Rachakonda PS, Bermejo JL, et al. Variants at the 9p21 locus and melanoma risk. BMC Cancer. Jul 2 2013;13:325. doi:10.1186/1471-2407-13-325
21. Jannot AS, Meziani R, Bertrand G, et al. Allele variations in the OCA2 gene (pink-eyed-dilution locus) are associated with genetic susceptibility to melanoma. Eur J Hum Genet. Aug 2005;13(8):913-20. doi:10.1038/sj.ejhg.5201415
22. Brown KM, Macgregor S, Montgomery GW, et al. Common sequence variants on 20q11.22 confer melanoma susceptibility. Nat Genet. Jul 2008;40(7):838-40. doi:10.1038/ng.163
23. Nan H, Qureshi AA, Han J. Melanoma susceptibility variants on chromosome 20q11.22 are associated with pigmentary traits and the risk of nonmelanoma skin cancer. Br J Dermatol. Feb 1 2010;162(2):461-3. doi:10.1111/j.1365-2133.2009.09579.x

Supplementary Table S2. Number of GEM participants successfully genotyped for each SNP and distribution of these participants by prognostic characteristics of primary melanoma tumor[1]

			Breslow thickness, mm					Ulceration			Mitoses			TIL grade			
			0.01-1.00	1.01-2.00	2.01-4.00	>4.00		Absent	Present		Absent	Present		Absent	Nonbrisk	Brisk	
Gene neighborhood	SNP	a/A	No. aa/aA/AA	No. aa/aA/AA	No. aa/aA/AA	No. aa/aA/AA	Total	No. aa/aA/AA	No. aa/aA/AA	Total	No. aa/aA/AA	No. aa/aA/AA	Total	No. aa/aA/AA	No. aa/aA/AA	No. aa/aA/AA	Total
ARNT	rs7412746	C/T	428/1023/713	122/264/194	55/134/85	26/69/46	3159	471/1100/788	48/102/71	2580	293/703/523	226/507/336	2588	127/257/181	317/762/552	70/184/123	2573
PARP1	rs3219090	A/G	208/968/960	52/248/284	26/126/116	22/51/65	3126	226/1053/1052	26/87/107	2551	142/690/665	110/454/498	2559	49/242/260	163/727/725	38/170/170	2544
PARP1	rs2695238	C/G	198/966/1009	52/237/294	28/119/127	23/52/67	3172	221/1039/1106	27/82/114	2589	139/683/707	109/441/518	2597	51/243/270	159/711/768	36/165/179	2582
NID1	rs3768080	G/A	528/1039/588	118/301/161	59/138/77	35/64/43	3151	564/1164/621	42/112/66	2569	377/745/392	231/535/297	2577	137/282/142	378/799/447	89/192/96	2562
NID1	rs10754833	C/T	546/1033/585	118/301/160	60/138/75	37/63/42	3158	579/1160/618	43/113/65	2578	390/741/392	233/537/293	2586	140/282/140	394/791/445	86/197/96	2571
CASP8	rs6735656[a]	G/T	156/846/1143	48/230/300	11/105/155	10/42/89	3135	178/906/1263	14/84/121	2566	116/591/804	76/403/584	2574	50/204/306	115/640/867	25/147/205	2559
CASP8	rs13016963	A/G	341/1003/827	89/272/221	30/129/114	25/49/68	3168	365/1062/934	32/107/83	2583	251/687/588	147/484/434	2591	90/248/225	245/742/645	61/177/143	2576
TERT	rs2242652	T/C	92/687/1297	21/167/363	7/90/163	7/44/88	3026	100/728/1423	9/67/134	2461	72/497/885	37/301/677	2469	23/163/351	74/517/957	12/116/241	2454
TERT	rs2853676	A/G	200/869/1101	51/233/300	29/107/138	16/40/84	3168	214/944/1206	24/75/123	2586	154/610/759	86/411/574	2594	49/236/279	150/633/851	41/147/193	2579
TERT	rs13356727	G/A	477/1082/621	110/278/201	56/134/83	27/77/38	3184	509/1161/706	41/114/69	2600	342/746/441	211/533/335	2608	130/270/165	333/822/489	87/183/114	2593
TERT;CLPTM1L	rs4975616	G/A	480/1079/550	114/282/166	55/136/69	29/77/32	3069	512/1160/618	42/117/48	2497	347/742/389	210/539/278	2505	130/274/144	333/818/431	91/185/84	2490
TERT;CLPTM1L	rs401681	T/C	532/1070/555	119/280/177	59/131/83	29/75/38	3148	564/1141/642	43/118/59	2567	383/737/394	227/526/308	2575	133/271/154	374/804/445	99/185/95	2560
SLC45A2	rs16891982	C/G	1/67/2098	1/19/563	0/9/266	2/1/140	3167	3/72/2283	1/6/217	2582	2/52/1466	2/26/1042	2590	2/23/533	1/45/1591	1/9/370	2575
SLC45A2	rs35391	T/C	1/37/2114	0/17/564	0/5/266	1/2/138	3145	1/45/2294	1/4/218	2563	1/30/1477	1/19/1043	2571	1/13/540	1/31/1592	0/5/373	2556
SLC45A2	rs26722	T/C	0/40/2107	0/13/564	0/4/265	0/3/136	3132	0/47/2286	0/5/212	2550	0/32/1474	0/20/1032	2558	0/13/539	0/34/1583	0/5/370	2544
SLC45A2	rs13289	G/C	288/993/881	74/282/221	45/141/84	20/61/61	3151	327/1096/928	38/99/85	2573	208/699/614	157/502/401	2581	93/275/194	215/755/655	54/166/159	2566
IRF4	rs12203592	T/C	112/713/1341	38/190/356	21/118/135	15/50/78	3167	143/803/1417	15/92/114	2584	86/514/924	72/383/613	2592	39/198/328	98/537/999	21/159/198	2577
IRF4	rs872071	A/G	470/1004/680	126/287/164	65/118/90	25/69/45	3143	502/1124/721	45/102/74	2568	310/729/474	237/500/326	2576	113/276/166	365/747/517	65/198/114	2561
TYRP1	rs1408799	T/C	199/868/1081	60/239/279	32/118/122	13/54/75	3140	205/962/1176	32/84/103	2562	132/603/775	106/446/508	2570	43/237/279	154/659/805	40/149/189	2555
TYRP1	rs2733832	C/T	330/1000/823	99/285/193	51/122/97	20/67/53	3140	359/1106/876	39/101/80	2561	223/699/584	175/513/375	2569	80/266/212	261/758/598	56/180/143	2554
MTAP	rs2218220	T/C	522/1027/615	111/282/190	61/154/59	35/69/38	3163	547/1144/667	46/114/62	2580	345/749/425	250/513/306	2588	108/280/172	390/778/465	92/198/90	2573
MTAP	rs1335510	G/T	349/963/840	67/261/252	43/136/92	24/64/53	3144	375/1063/907	26/106/88	2565	237/689/586	165/484/412	2573	75/242/242	264/739/618	61/184/133	2558
MTAP	rs7023329	G/A	502/985/659	108/289/185	59/145/67	35/68/37	3139	527/1115/701	39/122/58	2562	333/735/439	236/506/321	2570	114/264/181	370/767/483	80/203/93	2555
MTAP	rs10811629	G/A	371/985/800	84/258/243	39/147/87	29/66/47	3156	401/1077/877	31/112/78	2576	249/698/568	184/494/391	2584	85/254/224	283/746/598	64/184/131	2569
CCND1	rs11604821	G/A	267/1035/867	76/267/242	21/114/138	20/60/62	3169	278/1105/982	25/102/94	2586	177/750/596	127/458/486	2594	66/267/229	191/748/699	46/187/146	2579
CCND1	rs1485993	T/C	307/1033/821	84/263/236	20/115/131	19/65/57	3151	320/1092/938	29/106/88	2573	210/732/576	140/467/456	2581	74/257/226	221/746/663	53/191/135	2566
CCND1	rs11263498	T/C	293/1040/826	77/272/235	25/118/132	20/64/56	3158	310/1109/936	27/105/91	2578	201/741/572	137/474/461	2586	73/264/225	210/755/665	53/190/136	2571
*TYR***	rs1042602	A/C	282/1011/882	87/242/253	33/119/122	16/73/54	3174	298/1071/997	25/114/84	2589	200/697/630	123/492/455	2597	71/240/254	195/776/667	55/166/158	2582
TYR	rs10765198	C/T	266/950/956	76/238/274	40/124/110	15/66/59	3174	308/1035/1025	27/92/102	2589	181/669/676	154/463/454	2597	72/250/242	207/728/704	55/150/174	2582
TYR	rs1847142	A/G	302/988/879	84/243/256	45/126/104	20/67/54	3168	345/1068/950	33/100/90	2586	210/697/619	168/477/423	2594	85/258/220	232/755/649	60/156/164	2579
TYR	rs10830253	G/T	304/975/874	83/241/254	45/123/104	20/66/55	3144	346/1056/942	32/98/89	2563	211/688/613	167/472/420	2571	86/257/216	232/744/645	59/154/163	2556
ATM	rs12278954[b]	A/C	59/491/1624	17/128/441	5/67/200	2/38/102	3174	60/537/1769	7/50/166	2589	40/354/1130	27/235/811	2597	13/131/420	41/375/1219	13/80/290	2582
OCA2	rs1800407	A/G	21/371/1782	7/90/487	3/33/237	3/21/117	3172	26/382/1960	3/33/186	2590	14/264/1253	15/151/901	2598	10/91/463	14/257/1369	5/67/307	2583
OCA2	rs1800401	T/C	5/203/1970	5/46/536	0/29/246	0/14/128	3182	7/213/2154	1/20/202	2597	6/131/1395	2/102/969	2605	0/47/519	6/151/1487	2/33/345	2590
HERC2	rs1129038	G/A	113/749/1298	33/202/340	17/98/156	13/41/88	3148	115/800/1437	17/68/133	2570	75/529/914	60/342/658	2578	29/193/337	83/560/982	19/116/244	2563
HERC2	rs12913832	A/G	112/757/1302	36/201/347	17/100/158	14/39/89	3172	117/804/1446	17/69/137	2590	76/534/918	61/342/667	2598	27/196/340	87/562/991	19/116/245	2583
ASIP	rs17305657	C/T	25/421/1714	8/96/479	2/52/220	2/23/117	3159	26/449/1879	1/35/184	2574	16/302/1196	11/182/875	2582	4/101/457	17/300/1311	6/81/290	2567
ASIP	rs4911414	T/G	315/1020/832	74/292/221	39/125/110	15/67/61	3171	330/1130/905	35/101/88	2589	230/732/565	136/502/432	2597	74/271/220	231/785/621	60/172/148	2582
PIGU	rs910873	A/G	33/485/1656	3/124/461	5/62/208	1/28/112	3178	28/544/1800	3/39/181	2595	19/364/1147	13/220/840	2603	6/120/440	21/367/1253	5/94/282	2588
PIGU	rs17305573	C/T	27/410/1501	4/99/418	5/52/180	1/25/107	2829	25/456/1631	2/31/162	2307	18/304/1043	10/184/757	2316	5/101/394	18/309/1139	5/75/254	2300
NCOA6	rs4911442	G/A	65/582/1503	5/136/435	9/75/186	3/35/103	3137	55/639/1642	5/50/166	2557	40/421/1048	21/268/767	2565	12/139/404	40/441/1138	9/106/261	2550
MYH7B	rs1885120	C/G	28/445/1690	2/111/470	5/55/211	1/20/120	3158	24/496/1835	2/30/188	2575	18/326/1173	9/201/856	2583	5/108/447	17/330/1281	5/87/288	2568
LOC647979	rs1204552	A/T	14/372/1724	3/89/476	4/49/212	2/16/120	3081	17/404/1875	2/34/180	2512	12/270/1201	7/169/861	2520	7/89/459	10/273/1295	2/75/295	2505
MX2	rs45430	G/A	298/1046/820	57/256/269	39/117/119	20/64/58	3163	303/1112/944	28/95/98	2580	209/723/588	124/486/458	2588	68/249/244	208/770/654	57/182/141	2573
PLA2G6	rs6001027	G/A	245/919/897	74/231/237	30/100/125	19/64/48	2989	284/993/951	23/88/98	2437	186/649/604	120/438/447	2444	72/236/222	188/701/652	45/145/168	2429
PLA2G6	rs132985	T/C	440/1057/670	120/289/172	45/134/95	31/81/30	3164	494/1142/722	35/119/69	2581	314/740/463	216/526/330	2589	118/284/160	333/798/504	78/178/121	2574
PLA2G6	rs738322	G/A	448/1052/657	121/289/172	47/136/90	31/80/30	3153	500/1134/715	38/119/66	2572	319/733/460	220/525/323	2580	120/283/158	340/791/497	78/178/120	2565

Abbreviations: GEM, Genes, Environment and Melanoma; SNP, single nucleotide polymorphism; TIL, tumor-infiltrating lymphocyte; a, minor allele; A, major allele; No., number.

[1]Limited to individuals of European origin with incident invasive first- or higher-order primary melanoma who had their melanoma scored for the histopathologic variable of interest (i.e. Breslow thickness, ulceration, mitoses, or TIL grade).

[a]rs6735656 is a proxy for rs10931936 (r^2=0.965).

[b]rs12278954 is a proxy for rs1801516 (r^2=1.00).
